# An estimation and development model of tourism resource values at the township scale on Hainan Island, China

**DOI:** 10.1371/journal.pone.0262837

**Published:** 2022-01-24

**Authors:** Tongyan ZHANG, Yingjie WANG, Shengrui ZHANG, Yingying Wang

**Affiliations:** 1 College of Management, Ocean University of China, Qingdao, China; 2 Institute of Geographic Sciences and Natural Resources Research, CAS, Beijing, China; 3 University of Chinese Academy of Sciences, Beijing, China; 4 College of Resources and Environment, Shandong Agricultural University, Taian, Shandong, China; Northeastern University (Shenyang China), CHINA

## Abstract

The scientific evaluation of tourism resources provides the basic conditions for the rational development and utilization of tourism resources, which is significant for the sustainable development of tourism. On the basis of obtaining a large sample of tourism resource data, this study constructed an evaluation index system of regional tourism resources from four aspects: quantity, type, grade, and a combination of regional tourism resources, taking the township scale as the spatial evaluation unit. The combination weighting method was used to evaluate the grade of tourism resources in each township, and the hierarchical clustering method and spatial autocorrelation method were used to analyze the spatial patterns of the tourism resource values. On the basis of the evaluation’s results and spatial pattern analysis, this paper analyzed the influencing factors of tourism resource patterns and puts forward a tourism development model suitable for Hainan tourism development. The results showed that the overall value of tourism resources on Hainan Island was low, and the spatial pattern was obviously different, showing that the northern piedmont plain area was higher than the southern hilly area, and the east coast area was higher than the west coast area. Based on the analysis of quantity density, type abundance, grade advantage, and spatial combination, the tourism resource values of Hainan Island was divided into four combination areas: the first area was diversity and good combination, the second area was large quantity and good combination, the third area was diversity and high-quality resources, and the fourth area was large quantity. In addition, from the analysis of the spatial agglomeration effect, the phenomena of high and low agglomerations of tourism resource values were obvious. Finally, this paper puts forward a tourism development model for Hainan Island by grading, classification, and zoning. The results of this study can determine the time sequence and mode of regional tourism resources development and provide spatial implications and suggestions for regional tourism planning and management.

## 1. Introduction

The 2019 World Island Tourism Development Report revealed that the global tourism industry continues to grow and that island tourism is an important aspect of it [1]. For more than 40% of tourism destinations that are islands, it contributes more than 20% to their GDP [2]. Island tourism has become one of consumers’ favorite forms of tourism, which has promoted the development of world tourism [3]. Island tourism originated in Europe, America, the Caribbean, the Mediterranean Sea, Pacific Ocean, the Indian Ocean, and Southeast Asia. Many islands attract tourists from all over the world because of the sunny weather, waves, beaches, reefs, blue sky, white sails, and seafood, and islands have become a popular tourist destination [4]. Island tourism development in China originated in the late 1970s, mainly concentrating on Hainan Island, Shanghai, Hengsha Island, Putuo Island, Zhejiang, and in other coastal cities [5]. In 2010, the Chinese government began to promote the construction and development of the Hainan International Tourism Island and built Hainan into a world-class island leisure resort. As the only tropical island in China, Hainan Island occupies an important position in the international tourism market with its unique resource advantages and the policy support by the Chinese government. To a great extent, the development of tourism in a region depends on the abundance of resources and the scale and direction of development [6]. Therefore, tourism resources are not only the basic material condition of tourism development but also support regional tourism’s spatial competition [7, 8].

Scientific evaluation of tourism resources is an important basis and means for the rational development and utilization of tourism resources. It is the premise of tourism development and is of great significance to the healthy and sustainable development of tourism. Some early researchers evaluated the value of tourism resources such as landscape quality [9–12], currency value [13–17], and the suitability of resource and product transformation [18]. The evaluation of the potential for tourism resources development includes the attraction, accessibility, tourism facilities, and environmental quality of tourism resources [19–21]. Moreover, according to the value evaluation theory of tourism environmental resources, some scholars have introduced the travel cost method (TCM) and conditional value method (CVM) to evaluate environmental resources [22–24].

The above research was aimed at individual or individual tourism resources, but those with a high value of tourism resources may not be able to form at scale. Only when they are concentrated in a certain region (i.e., have a certain abundance and density) and have a coordinated layout and combination of various types of resources can they form a certain scale of development. Therefore, a region’s tourism resources exist in the form of tourism resource groups, and the rational development and utilization of tourism resources are influenced by their quantity, type, abundance, hierarchy, spatial combination, and external environment [25]. Scholars have also conducted relevant studies on the evaluation of regional tourism resources clusters such as comprehensively evaluating the value of a region’s tourism resources from the aspects of total tourism resources, monomer density, type abundance, reserve abundance, average quality, and excellent monomer quantity [26–32]. Some scholars believe that stakeholder cooperation plays a leading role in tourism development and planning [33]. Tourism development is an important method for local governments to develop the local economy, but it is necessary to attract tourists according to the corresponding basic conditions. For those regions with competitive conditions in the process of regional tourism development, the largest problem lies in how to transform their relative resource advantages into competitive advantages, and the introduction of the concept of industrial clusters will help to promote this process [34]. There are many evaluation methods for tourism resources including the evaluation of resources, themselves, and the overall evaluation of regional divisions. The former is the value of determining the location that is suitable for tourism or entertainment development, while the latter regards choosing the key development locations from several departments [35].

From previous studies, it can be found that scholars have begun to pay increasing attention to the regional evaluation of tourism resources. At present, most of the studies are carried out at the county level, which mainly analyzes and evaluates the value of tourism resources from three aspects: quantity, type, and grade, while there are few studies on the comprehensive evaluation of the value of tourism resources at the township level in a region. Towns (streets) are the basic units of Chinese administrative divisions. Exploring the distribution law of tourism resources at this scale has important reference values for creating regional tourism development plans, and it can better reflect the challenges in the process of regional tourism development. The value of tourism resources in villages and towns should be paid more attention. If this value is underestimated or overestimated, it will create misleading policies for the protection and development of regional tourism resources. Therefore, for the sustainable development of regional tourism, it is of great significance to evaluate and develop tourism resources in villages and towns. In this study, by constructing a Hainan Island tourism resources spatial database, taking the township scale as the evaluation unit, the value index system of township tourism resources was constructed from four aspects: quantity, type, quality, and spatial combination. The weight of each index value was calculated by subjective and objective combination weighting methods, and the noumenon value of tourism resources in 184 townships was evaluated. On the basis of the evaluation’s results, using hierarchical clustering analysis and spatial autocorrelation analysis, this paper analyzed the spatial pattern and influencing factors of tourism resource values and puts forward a tourism development model of Hainan Island, providing a reference for regional tourism development and management.

## 2. Research region

**Fig 1:**
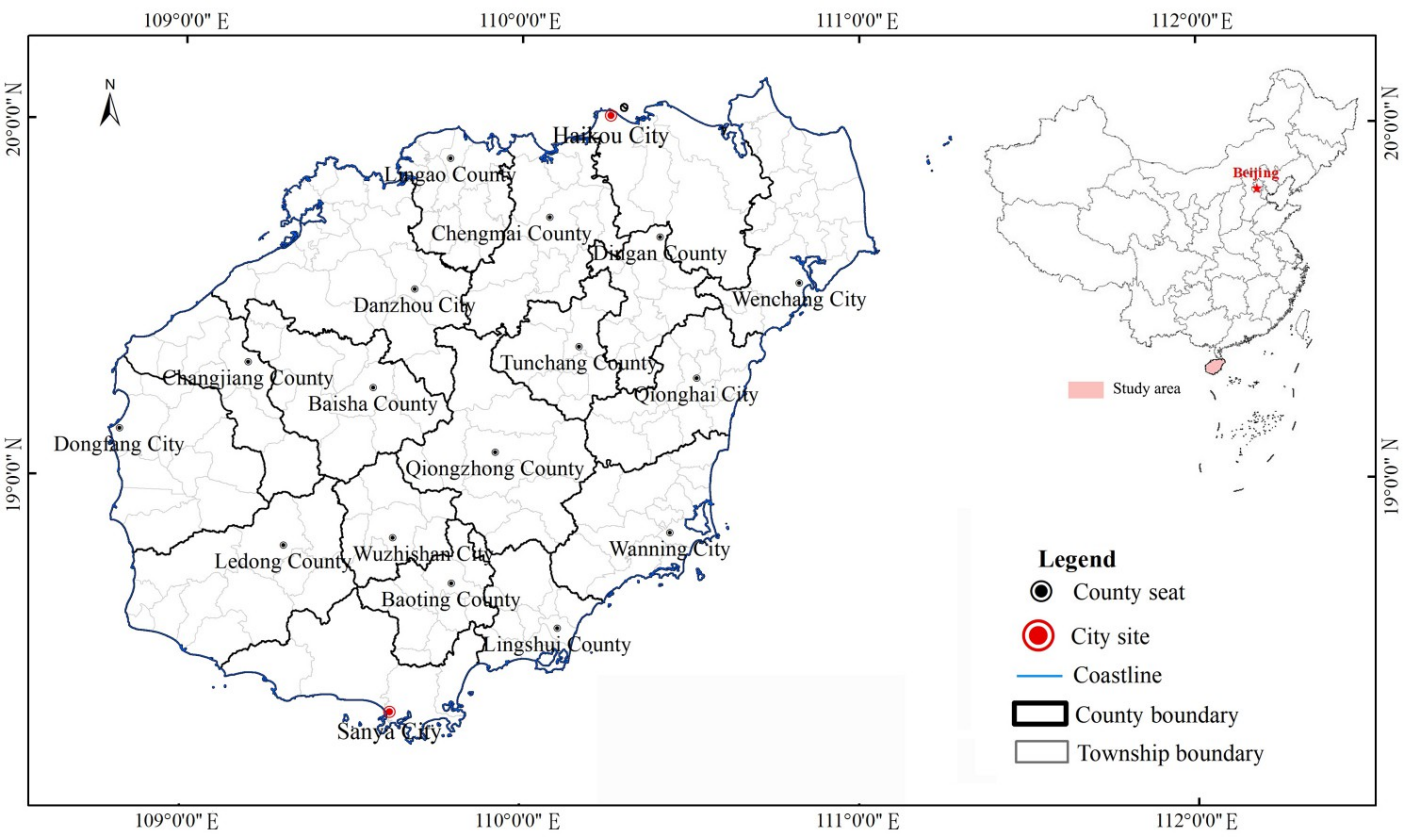
Location of the study area (The base map was published by Hainan Administration of Surveying Mapping and Geoinformation: Http://hism.mnr.gov.cn/sjkf/bzdt/, which was open and free with no copyright disputes).

Hainan Province is located in the southernmost part of China, which has a total land area of 35,400 km^2^ (mainly comprising Hainan Island, Xisha Islands, Zhongsha Islands, and Nansha Islands). Hainan Island is the second largest island in China after Taiwan Island, with an area of 33,900 km^2^. Guangdong Province borders the Qiongzhou Strait in the north; Vietnam is across the sea to the west; the Philippines, Brunei, Indonesia, and Malaysia are to the east; the South China Sea lies to the south. Hainan Island is oval in plane; the highest point is Wuzhi Mountain in the middle, and it descends to the periphery step by step. It consists of mountains, hills, terraces, and plains that surround the mountains in the middle part of China. The outermost circle of the zonal structure on Hainan Island is the coastal plain belt around the island, which consists of a coastal plain, sandbar plain, lagoon plain, and delta plain. The climate belongs to a tropical maritime climate, which is warm and hot all year round, with abundant rainfall, obvious dry and wet seasons, frequent typhoon activities, and diverse climate resources; it is known as the treasure house of tropical resources in China. The island has undulating terrain and crisscrossing rivers, forming abundant forest layers and geomorphic units. Hainan Island, with its unique geographical location, climatic characteristics, and good ecological environment, is a high-quality tourism space suitable for all seasons, which is very attractive to the market, and it is one of the key tourist areas in China. In this study, 184 towns and districts administrative units on Hainan Island were selected as the research objects: Haikou city, Sanya city, Danzhou city, Wuzhishan city, Wenchang city, Qionghai city, Wanning City, and Dongfang city; Ding’an County, Tunchang County, Chengmai County, Lingao County, and Baisha Li Nationality (Fig 1).

## 3. Data and methods

### 3.1. Data sources and preparation

Tourism resource data for Hainan Island were obtained from five sources: tropical forest tourism planning texts of Hainan Province, the database of the second census of geographical names, high-resolution remote sensing images, the official tourism website of Hainan, and a field survey conducted in 2018. The field survey covered the entire island and collected 10,260 spatial points of data, including data on developed, developing, and undeveloped tourism resources. The survey provided the location, type, nature, and characteristics of tourism resources, the surrounding environment, and attribute information on the protection and development conditions. Among the investigated attributes, only the type and value attributes of tourism resources were used in this study. According to China’s national standard, “Classification, Investigation and Evaluation of Tourism Resources” (GBT 18972–2017) and on the basis of Hainan’s natural environmental characteristics, tourism resources were divided into nine categories: geological landscapes; water landscapes; biological landscapes; astronomical phenomena and meteorological landscapes; buildings and facilities; ruins and remains; human activities; tourism commodities; ocean and coastal landscapes. These categories can be further divided into 25 subtypes and 135 fundamental types. We asked local experts, tourism experts, geography experts, and government personnel to score the tourism resource value. The evaluation results were divided into five grades (Table 1).

**Table 1:** Evaluation grades of tourism resources.

Score Interval	Grade of Tourism Resources
≥90	Level five
75–89	Level four
60–74	Level three
45–59	Level two
30–44	Level one

### 3.2. Tourism resources value indicator system development

The abovementioned tourism resource grades are only for the evaluation of individual tourism resources, and it is also necessary to evaluate the value of tourism resource groups within a certain space a second time. Within a certain spatial scope, tourism resource groups are characterized by a large number of individual units, various types, different grades, and various spatial combinations. Based at the township scale, this paper constructed the quantity density, type diversity, grade superiority, and spatial combination to express the quantitative, type, grade, and combination characteristics of tourism resources, and it comprehensively reflects the self-value of tourism resources groups with a certain spatial scale. The meanings and calculation formulas of specific indicators are as follows.

#### 3.2.1. Quantity density

Quantity density refers to the quantity of tourism resources per unit area of a certain spatial unit. When there are more tourism resources in a certain scale space, the scale of tourism resources will be larger and the value of tourism resources will be greater.


Sr=mA (1)

In the formula, *S*_*r*_ is the quantitative density, *m* is the total number of regional tourism resources, and *A* is the area.

#### 3.2.2. Type diversity

Type diversity refers to the type diversity of tourism resources in a space unit. Therefore, the richer the types of tourism resources, the more diverse the tourism landscapes, providing more sightseeing opportunities for tourists, a higher value for the tourism resources.


$$R_{r}=n⁄N$$ (2)

For the proportion of the fundamental types of tourism resources in villages and towns, in the formula, *R*_*r*_ is the type abundance, *n* is the number of fundamental types in the region, and *N* is the total number of fundamental types in the province.

#### 3.2.3. Grade superiority

Grade advantage refers to the position of excellent tourism resources in the same spatial unit and the same type of tourism resources group, indicating the dominant position of this type of tourism resources in the tourism resources group. In a certain spatial unit, the better the tourism resources, the better the advantages and the higher the value of township tourism resources.


Dr=∑i=15wi×mim (3)

For the proportion of tourism resources at all levels in the region, in the formula, *D*_*r*_ is the quality proportion, *i* is the level of tourism resources, *w*_*i*_ is the weight of level *i*, (level 5 is 1.0, level 4 is 0.7, level 3 is 0.5, level 2 is 0.2, and level 1 is 0.1), *m*_*i*_ is the number of tourism resources of level *i*, and *m* is the total number of regional tourism resources. The weight of each level was determined through expert scoring.

#### 3.2.4. Spatial combination

Spatial combination is a highly dependent and indivisible combination of tourism resources formed by several individual resource components with similar geographical positions and different resource levels according to certain landscape structures and functions; this shows the combination rule in type, quality level, and regional space. The better the degree of integration, the higher the grade allocation, the more individual types, the more coordinated the proportion of tourism resources in the region, the closer the links among tourism resources, and the greater the attraction to tourists. The index of combined regional tourism resources reflects the coordination degree of a combination of tourism resources under a certain distance threshold. The more diversified the combination types, the higher the value of regional tourism resources. This study mainly referred to the network in graph theory to express the combination relationship among different types of tourism resources. Under a certain distance threshold, tourism resources of different categories are expressed as a binary group G = (V, E), where V is the set of tourism resources points, and E represents the set of the connecting edges of different categories of tourism resources, where each edge is formed by connecting two points in V.


$$C_{r}=\frac{2\left|E_{d}\right|}{\left|V\right|\left(\left|V\right|-1\right)}$$ (4)

Here, *C*_*r*_ is the combination degree of resources; *E*_*d*_ is the number of connecting edges for threshold distance *d*; *d* is the distance threshold, the median of the nearest distances of tourism resources in a certain area; *V* is the number of edges in the network. The larger the *C* value, the more coordinated the tourism resources are in the regional distribution.

### 3.3. Evaluation method for tourism resource values

#### 3.3.1. Standardization of the evaluation index

Because each index has different characteristics and dimensions, it cannot be calculated directly; thus, it was necessary to carry out dimensionless processing on each index value to obtain a standardized index data matrix. Let the sample set of index value be x_*ij*_(m*n): x_*ij*_ represents the *j*-th index of sample *i*, m is the number of samples, and *n* is the number of indexes. Formula (5), which calculates the forward index, and Formula (6), which calculates the reverse index, are as follows [36, 37]:

$$x_{ij}^{\prime }=\frac{x_{ij}-\text{min}\left(x_{j}\right)}{\text{max}\left(x_{j}\right)-\text{min}\left(x_{j}\right)}$$ (5)

$$x_{ij}^{\prime }=\frac{\text{max}\left(x_{j}\right)-x_{ij}}{\text{max}\left(x_{j}\right)-\text{min}\left(x_{j}\right)}$$ (6)

In the formula, $x_{ij}^{\prime }\;$ represents the standardized value of the j-th index of the i-th sample, *x*_*ij*_ represents the j-th index value of the i-th sample, and max(*x*_*j*_) and min(*x*_*j*_) reprent the maximum and minimum values of the j-th index.

#### 3.3.2. Determination of the evaluation index weight

The analytic hierarchy process (AHP) method is a comprehensive evaluation method combining qualitative and quantitative analyses; it has high reliability and accuracy but is easily affected by subjective factors [38]. The entropy weight method objectively determines the weight according to the distribution information of data, but it is greatly influenced by data. Therefore, this study integrates subjective and objective weighting methods to calculate the weight, avoid the deviation of the single weighting method, and to improve the objectivity, accuracy, and rigor of the comprehensive measurement of the evaluation index system [37].

1. Analytic hierarchy process (AHP)

The core idea of the AHP method is to decompose complex evaluation problems, form a multi-level and multi-factor structure, and compare and judge the importance between two indicators. By constructing a judgment matrix, calculating the maximum eigenvalues and the corresponding eigenvectors of the matrix, and calculating the index weight subset of the single layer through the single layer consistency test, the weights of different indexes are finally obtained [39].

2. Entropy weight method

In information theory, “entropy” is used to measure the disorder of a system. The smaller the value, the more information is contained in the index. The concept of entropy is used for comprehensive evaluations. The smaller the entropy value of an index, the greater the information it expresses and the higher the importance it plays in the index evaluation system, so the greater the weight it should give [39]. In this study, “entropy” was used to determine the objective weight; that is, the weight value was determined according to the difference degree of each index. The entropy value was calculated using the normalized result of Formula (5), and the weight was determined. The specific calculation formulas are shown in Formulas (7) and (8), respectively.

$$H_{i}=-k\sum_{j=1}^{n}p_{ij}\text{ln}p_{ij}$$ (7)

$$w_{j}=\frac{1-H_{i}}{m-\sum H_{i}}$$ (8)

In the formula, $p_{ij}=\frac{y_{ij}}{\sum_{j=1}^{n}y_{ij}}$, *k* is a constant, and k=1lnn.

3. Combination weighting method

The weights are combined using the sum of weights obtained by the AHP and entropy weight methods, and the calculation formula is as follows.


$$W_{j}=\frac{w_{1j}\cdot w_{2j}}{\sum_{j}^{n}w_{1j}\cdot w_{2j}}$$ (9)

In the formula, *W*_*j*_ is the combination weight of j index, *w*_1*j*_ is the weight calculated by the AHP, and *w*_2*j*_ is the weight calculated by the entropy weight method.

#### 3.3.3. Calculation of comprehensive evaluation index

After obtaining the index value and combination weight, the value of tourism resources is calculated by linear weighting method [40].


$$Z_{i}=\sum_{j}^{n}W_{j}\cdot x_{ij}^{\prime }$$ (10)

In the formula, *Z*_*i*_ is the value score of the i-th township tourism resources, and *w*_*j*_ and $x_{ij}^{\prime }$ are the same as Formula (8) and Formulas (5) and (6).

### 3.4. Hierarchical clustering analysis

In order to measure the differences among tourism resources values in different dimensions, hierarchical clustering algorithms based on cosine distance were introduced to divide the value combination of tourism resources in each township. Cluster analysis is an analysis process in which multiple objects without obvious classification characteristics are divided into multiple clusters according to certain similarities [41]. At present, there are many clustering algorithms and techniques such as K-means clustering, DBSCAN clustering, compressed hierarchical clustering, and EM clustering. The essence of hierarchical clustering analysis is to establish a classification method that clusters the most similar objects together according to the proximity between cases and variables. Its advantage is that the clustering algorithm is simple, fast, and widely used, and the distance measure is not strictly selected [42]. A hierarchical clustering algorithm is an iterative solution method that includes the following steps.

Step 1. Select the number of clusters to start data clustering;
Step 2. Each data point in the original data is regarded as a class, and then the ranging standard between the two classes is selected;
Step 3. Combine the minimum cosine distance of the two clusters into one cluster and iterate continuously until all data points are combined into one cluster.

The expression for calculating cosine distance is as follows.


$$C_{ij}=\text{cos}\;a_{ij}=\frac{\sum_{k=1}^{n}x_{ki}x_{kj}}{\sqrt{\sum_{k=1}^{n}x_{ki}^{2}\sum_{k=1}^{n}x_{kj}^{2}}}\;,\;d_{ij}=1-C_{ij}$$ (11)

### 3.5. Spatial autocorrelation analysis

Spatial autocorrelation analysis is an effective method to test the aggregation and anomaly of spatial unit attributes including two measurement methods: Moran’s I index in global space and Moran’s I index in local space. Global spatial correlation measurement studies the global correlation, spatial distribution pattern, and significance of all spatial objects in a region. The calculation formula is:

$$I=\frac{\sum_{i=1}^{n}\sum_{j=1}^{n}W_{ij}\left(x_{i}-\bar{x}\right)\left(x_{ij}-\bar{x}\right)}{\sigma^{2}\sum_{i=1}^{n}\sum_{j=1}^{n}W_{ij}}$$ (12)

In the formula, *x*_*i*_ is the score for the value of tourism resources in the i-th township, n is the number of townships, $\bar{x}$ is the average value score of tourism resources, *σ*^2^ is sample variance, and the spatial weight matrix, *W*_*ij*_, is a binary adjacency matrix. The range of the global Moran index is (–1,1). If the Moran index is close to 0, it shows that many of the values of the regional tourism resources are randomly distributed and there is no spatial autocorrelation. If the value is positive and close to 1, it means that there is a positive spatial correlation between the values of adjacent areas, and if the value is negative and close to –1, it means that there is a negative spatial correlation between the values of adjacent areas.

The local Moran index further reveals the clustering characteristics of local regional units in adjacent spaces. *I*_*i*_ measures the degree of association between spatial unit *i* and its adjacent spatial unit ontology value, and its calculation formula is as follows:

$$I_{i}=\frac{\left(x_{i}-\bar{x}\right)}{\sigma^{2}}\sum_{j=1}^{n}W_{ij}\left(x_{j}-\bar{x}\right)$$ (13)

The local Moran index is positive (negative), indicating that similar (different) attribute value elements are adjacent in space, and the greater the absolute value, the higher the degree of closeness. The agglomeration types are divided into four types: H–H, H–L, L–L, and L–H agglomeration.

## 4. Evaluation and spatial pattern analysis of tourism resources at the township scale in Hainan Island

### 4.1. The value grade distribution of tourism resources

**Fig 2:**
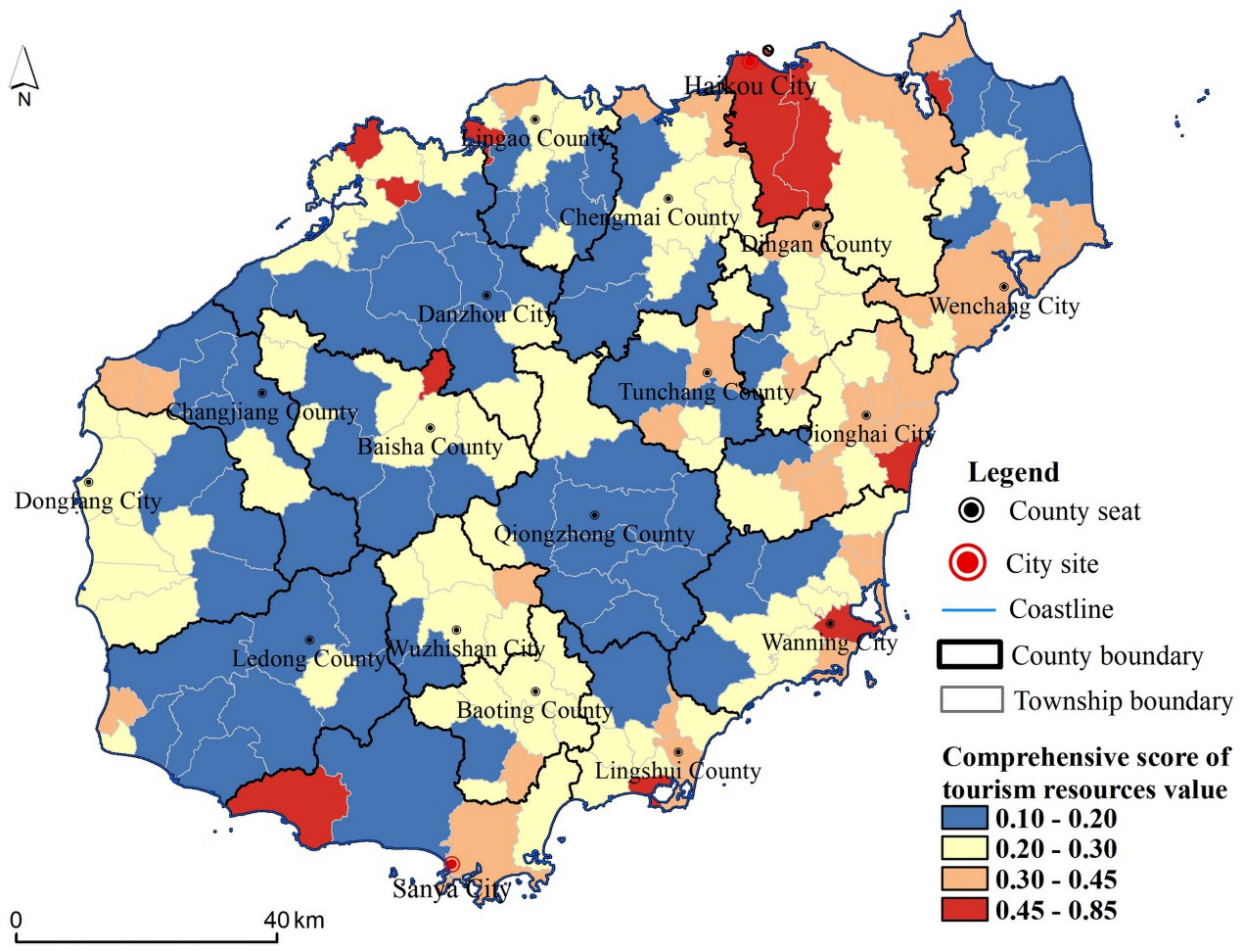
Distribution of tourism resources values at the township scale on Hainan Island (The base map was published by Hainan Administration of Surveying Mapping and Geoinformation: Http://hism.mnr.gov.cn/sjkf/bzdt/, which was open and free with no copyright disputes).

According to the evaluation index system and comprehensive evaluation method, taking 184 villages and towns in Hainan Island as tourism development units, the tourism resources value of each village and town in Hainan Island was calculated. Using the natural breakpoint method, the value of the tourism resources was divided into four grades: the highest value was 0.45–0.85, the higher value was 0.3–0.45, the lower value was 0.2–0.3, and the lowest value was 0.1–0.2. The differentiation pattern of the tourism resources values of villages and towns in Hainan Island was obtained (Fig 2). Fig 2 shows the sporadic distribution of the highest value, involving 11 townships, mainly distributed in four townships in the north of the city, one township on the west coast, three townships on the east coast, and Sanya in the south. The higher value was scattered and the number was relatively small, including 32 townships that are concentrated in the Qionghai area in the north. The lower values were scattered and large in number, involving 80 townships, mainly distributed in the central mountainous areas and southwest coastal areas. The lowest value involved 61 villages and towns, mainly concentrated in the central mountainous areas and southwest coastal areas and also concentrated in Tunchang in the north.

### 4.2. The value combination distribution of tourism resources

**Fig 3:**
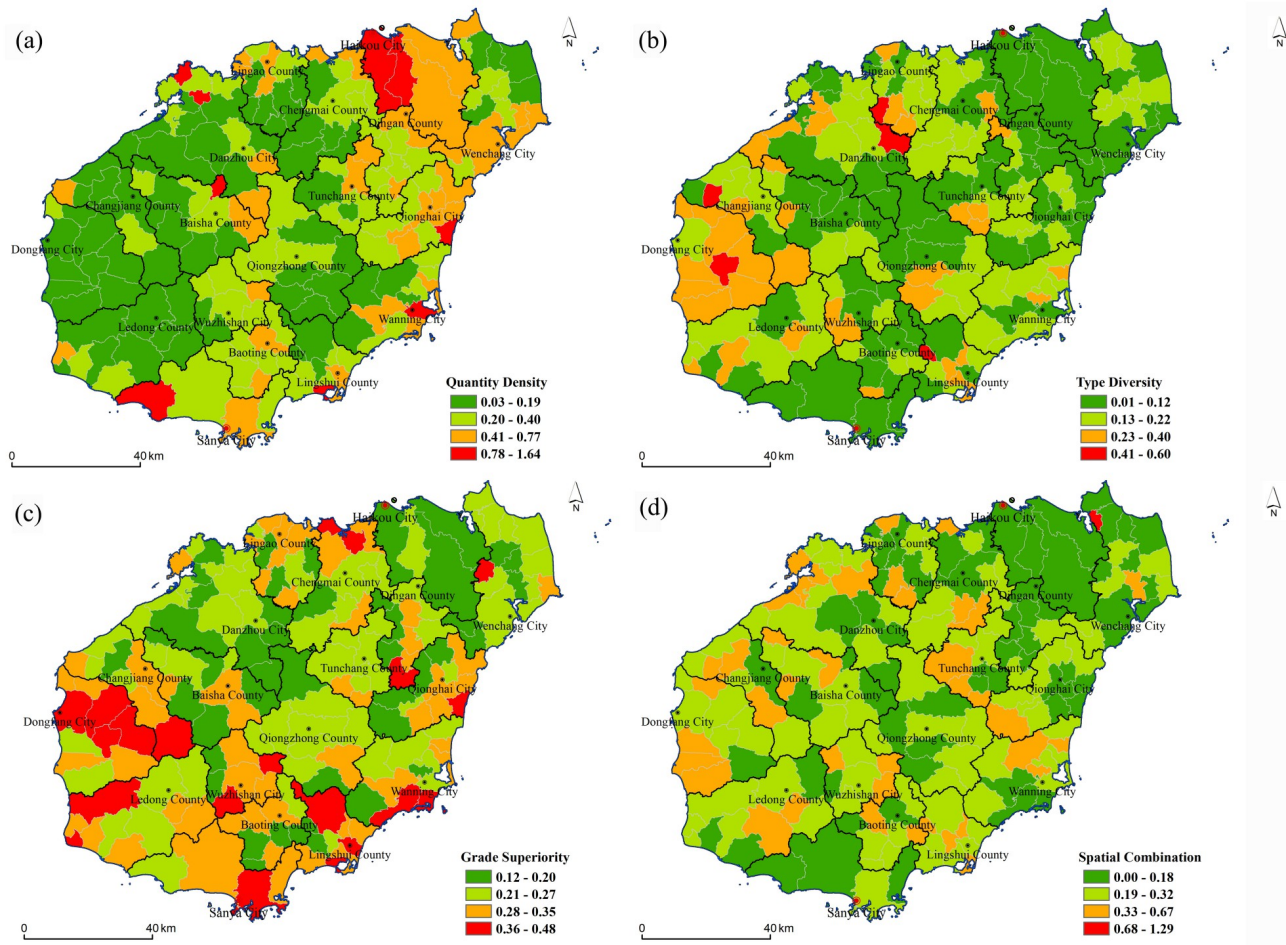
Spatial distributions of the (a) quantity density, (b) type diversity, (c) grade superiority, and (d) spatial combination (The base map was published by Hainan Administration of Surveying Mapping and Geoinformation: http://hism.mnr.gov.cn/sjkf/bzdt/, which was open and free with no copyright disputes).

**Fig 4:**
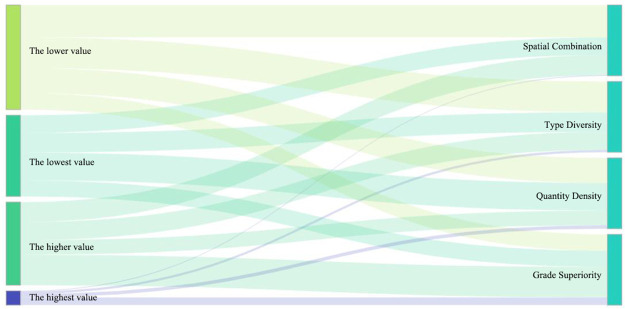
Comparison of the number of villages and towns with different dimension indicators of tourism resources values.

The spatial difference patterns of the four dimensions were compared: quantity density, type diversity, grade superiority, and spatial combination (Fig 3). Fig 3 shows that the spatial differentiation patterns of the four dimensions showed an obvious differentiation patterns of high value areas and low value areas. There were obvious regional differences and concentrated distributions between high and low value areas of quantitative density. The high-value areas were distributed in the north of the Haikou provincial capital city and the southeast coastal area of Sanya, while the low-value areas were distributed in the east and southwest coastal area of Ledong City. There were also obvious regional differences in the distribution of high-value areas and low-value areas that were relatively concentrated. The high-value areas were distributed in Dongfang City on the west coast, while the low-value areas were distributed in Haikou and Anding in the north, Sanya in the south, Baisha, and Qiongzhong mountain in the middle, which were just opposite to the distribution trend of quantity density, indicating that although there are many tourism resources in these areas, the types are relatively single. Grade superiority and spatial combination were scattered, but the differentiation patterns of high and low dominance values were obvious. The areas with higher dominance values were mainly distributed in the southern coastal areas, while the overall integration level was not high and the spatial differentiation pattern was not obvious. Generally speaking, from the comparison of the number of villages and towns in different dimensions, the low values of number density, richness, and combination degree were greater than the high values, while the high values of dominance degree were greater than the low values (Fig 4).

**Fig 5:**
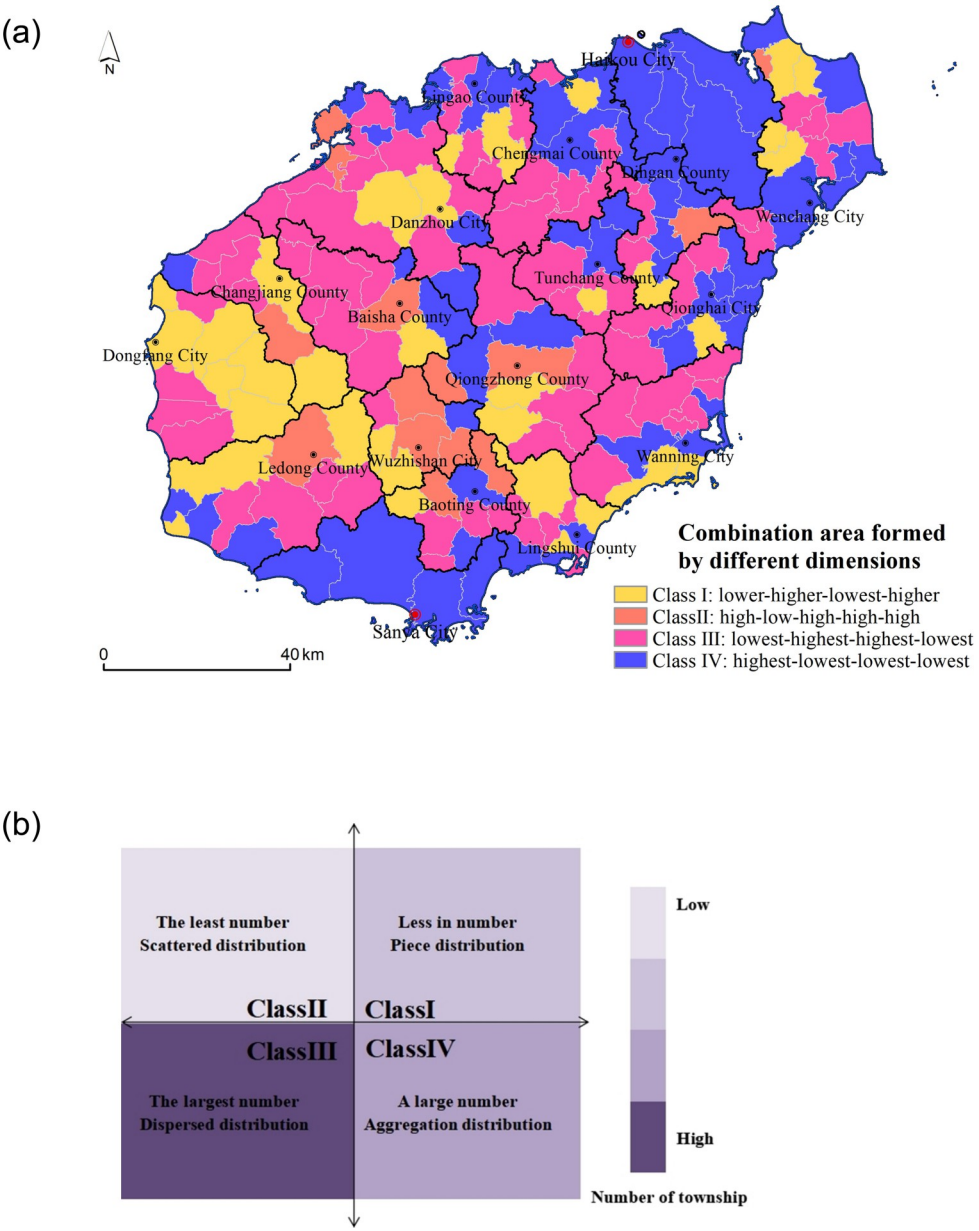
Combined distribution of various dimensions of tourism resources values (The base map was published by Hainan Administration of Surveying Mapping and Geoinformation: Http://hism.mnr.gov.cn/sjkf/bzdt/, which was open and free with no copyright disputes).

According to the results of each dimension index value, four different classification results were obtained by hierarchical clustering analysis. Class I: lower–higher–lowest–higher, which indicates that the total amount and quality of resources of this kind were small, but the types were diverse and the resource combination was good. Class II: high–low–high–high–high, which shows that the total amount of tourism resources in this area was large, the quantity of high-quality resources was relatively large, and the resource combination was also the best, but the type was relatively single. Class III: lowest–highest–highest–lowest, which shows that the total amount of this kind of tourism resources was small, the combination was poor, but there were many kinds and the quality of the resources was high. Class IV: highest–lowest–lowest–lowest, which shows that the total amount of this kind of tourism resources was relatively high, but there were few high-quality resources, single types, and poor combinations (Fig 5). It can be seen from Fig 5 that the first type of resources were diverse and well combined, mainly distributed in the central mountainous area, involving 34 towns and villages, accounting for 18% of the total number of towns and villages on Hainan Island. The second category of resources was large in quantity and well combined, scattered in the Wuzhishan area, involving 14 townships, accounting for 8%. The third category of diversified high-quality resources was scattered in the southern region, involving 77 towns and villages, accounting for 42%. The fourth region with higher total resources was mainly distributed in Haikou city in the north and the Sanya tourist area in the south, involving 59 townships, accounting for 32%. Altogether, the spatial patterns of township tourism resources on Hainan Island could be divided into four combined areas: The first category was diversified and well combined, with the characteristics of small quantity but concentrated distribution. The second type was of large quantity and well combined, which was characterized by small quantity and scattered distribution. The third category was diversified high-quality resources, which have the characteristics of large quantity but a scattered distribution. The fourth was a large number and concentrated distribution.

### 4.3. The value agglomeration distribution of tourism resources

According to the analysis using GeoDa software, the Moran’s I index value of the tourism resources value of 184 villages and towns in Hainan Island was 0.237, and the normal statistic z-value of Moran’s I index value was greater than the critical value of the 0.05 confidence level 1.96. The Moran’s I index was at a significant level of 0.1%, and the tourism resources value of 184 villages and towns had significant global spatial autocorrelation characteristics. The spatial distribution of tourism resources value on Hainan Island presents the spatial agglomeration of similar values, rather than a completely random state, that is, the spatial characteristics of high-value agglomeration and low-value agglomeration of a tourism resources value index.

**Fig 6:**
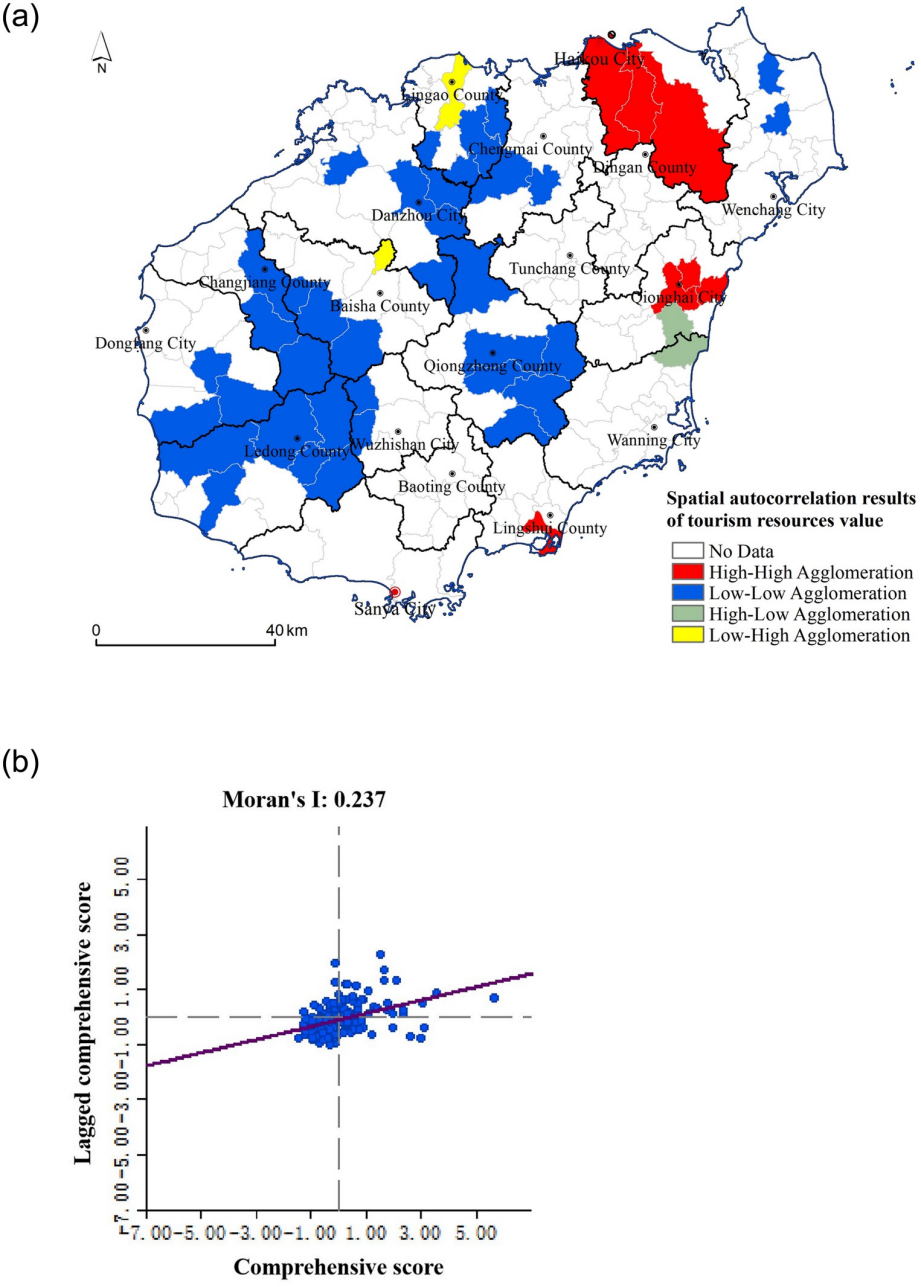
Moran’s I scatter plot and an LISA aggregation map of tourism resources value (The basemap was published by Hainan Administration of Surveying Mapping and Geoinformation: Http://hism.mnr.gov.cn/sjkf/bzdt/, which was open and free with no copyright disputes).

The results of Moran’s I show that the value of tourism resources on Hainan Island shows significant spatial correlation as a whole, but it fails to show where there are high-value agglomeration or low-value agglomeration. In order to further study the local spatial aggregation characteristics of tourism resources value on Hainan Island, the Moran scatter plot was selected to draw an LISA aggregation graph to describe the local spatial heterogeneity characteristics of the tourism resources value (Fig 6). As can be seen from Fig 6, the spatial aggregation types of the value index of each township unit were divided into four categories that corresponded to the four quadrants in the figure: The first quadrant is the “H–H” aggregation area, and the ontology value of the township unit itself and its surrounding areas was high, and the spatial difference between them was small and positively correlated. The second quadrant is the “H–L” gathering area, where the unit value of the villages and towns was higher than the surrounding areas, and there was a large spatial difference among them, showing a negative correlation. The third quadrant was the “L–L” gathering area, and the value of the township unit itself and its surrounding areas was low, and the spatial difference among them was small and positively correlated. The fourth quadrant is the “L–H” gathering area. The value of the township units was low, but the value of the surrounding areas was high. There was a large spatial difference and negative correlation between them. There were only 3–4 townships in the second and fourth quadrants, and there were two differentiation modes in the first and third quadrants: high concentration (HH) and low concentration (LL).

The high–high agglomeration regions were mainly distributed in northern Haikou and Qionghai, including eight towns such as Dongshan town, Zuntan town, Hongqi town, Penglai town, and Huiwen Town. These places have a good foundation for tourism and superior locations, which will become international tourism centers and play an important role in the construction of the 21st Century Maritime Silk Road. Although Sanya is an important city for tourism development, its tourism resources value was at a high level, while the neighboring Ledong tourism resources value index was relatively low, so the resource value in the southern region did not seem to be gathered at a high level.

The low–low agglomeration regions were mainly distributed in the central and southern mountainous areas, such as Qiongzhong, Wuzhishan, and Ledong, including 35 townships such as Guoli town, Baoyou town, Wanchong town, Maoyang town, and Yingen town. These places belong to the urban–rural fringe, and tourism is relatively underdeveloped, but the potential for development of the tourism resources is large.

The agglomeration phenomena of high–low agglomeration regions and low–high agglomeration regions were not significant, and they were two types of agglomeration between high–high agglomeration areas and low–low agglomeration areas. Long Fu Township and Zuntan town, which had high tourism resources values, were not in the same quadrant, because the adjacent areas to Zuntan town, such as Dongshan town and Hongqi town, were also high-value areas, that is, high-value areas surrounded by high-value areas, reflecting the spatial dependence of tourism resource values in geographical spatial distributions. However, Nanfeng town and Yaxing town, which are adjacent to Long Fu Township, had lower values, that is, the high and low gathering areas were surrounded by areas with lower noumenon values, which reflects the heterogeneity of tourism resources values in geographical spatial distributions.

**Fig 7:**
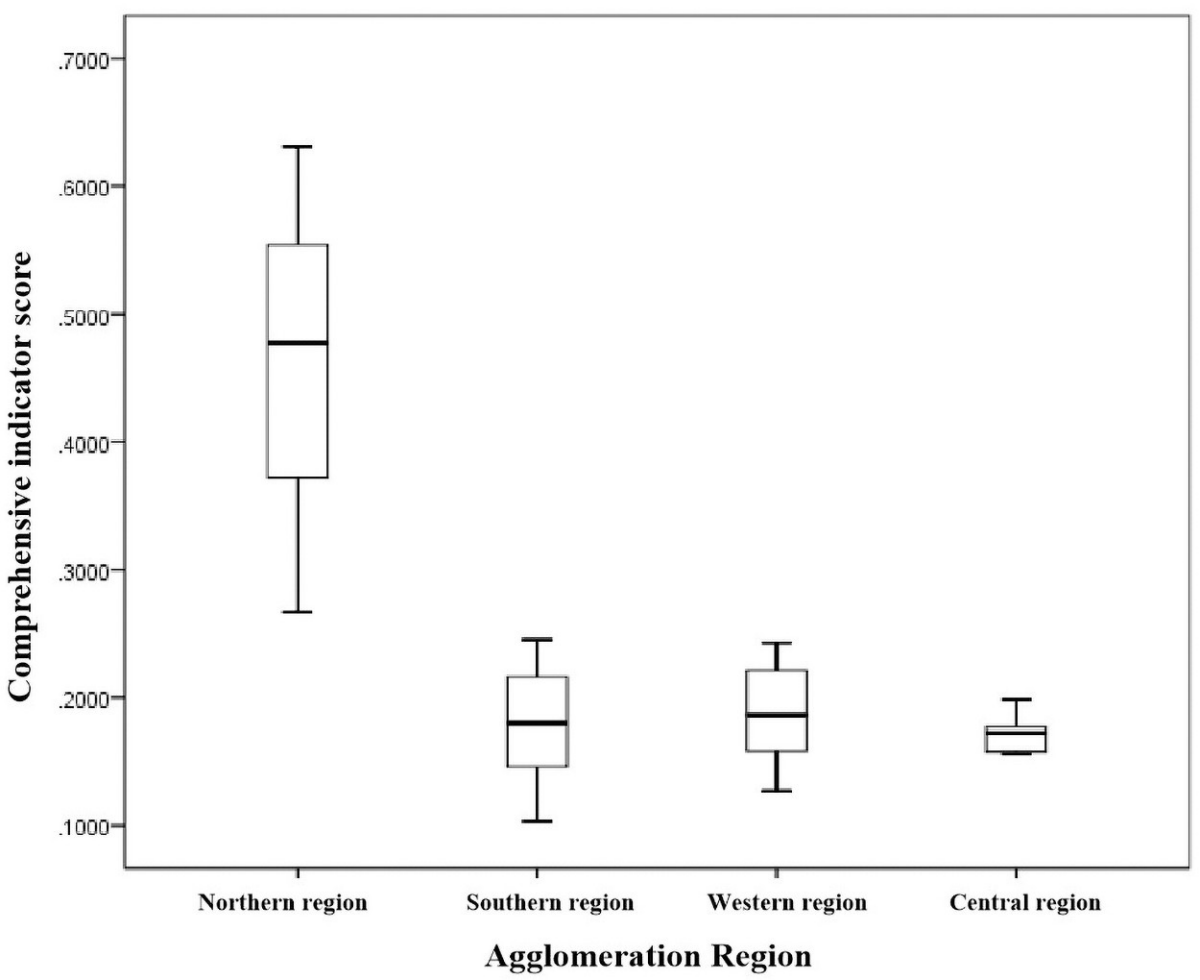
Comprehensive score box diagram of tourism resource values on Hainan Island via agglomeration regions.

The results of high concentration and low concentration were divided into four concentrated areas of tourism resources: north concentrated area (Haikou area), south concentrated area (Ledong–Baisha area), west concentrated area (Danzhou–Lingao area), and the central concentrated area (Qiongzhong area). The comprehensive score of the tourism resources values of each gathering area is presented in a box diagram (Fig 7). It can be seen from the figure that, according to the overall data distribution trend, the score value in the northern region was the highest, followed by the western and southern regions, and the distribution interval of the central region was the lowest. The value of the tourism resources on Hainan Island shows a differentiation pattern from the northern, western, southern, and central plains to hilly areas.

### 4.4. Factors influencing the spatial pattern of tourism resources values

#### 4.4.1. Natural environment

Terrain, climate, hydrology, and other natural factors have an important impact on the distribution of Hainan Island’s tourism resources. As far as natural tourism resources are concerned, complex topography and diverse hydrometeorological conditions have created various landscapes for tourism. In addition, natural factors have a great influence on the distribution of humanistic tourism resources. Natural factors determine regional accessibility and communication efficiency with the outside world, which leads to different cultures, architectural styles, and facilities. The spatial patterns of tourism resources with different qualities on Haikou and Lingshui are a good example. Haikou has a remarkable cluster of high–high building facilities, while Lingshui has a cluster of low–low forest landscapes. One reason for the difference may be that Lingshui is close to the east coast, and there is complex mountainous and hilly terrain in Lingshui that creates various vegetation landscapes, which leads to the high-level cluster of Lingshui forest landscapes and creates local traditional culture and folk customs. Therefore, the spatial differences in the quality of island tourism resources may be caused by natural factors such as topography, climate, and hydrological conditions.

#### 4.4.2. Cultural diversity

Regional cultural diversity is another factor that affects the spatial difference in tourism resource quality on Hainan Island. Due to the geographical location and historical factors, the cultures and customs of residents living in the same area may be similar. If there are obvious cultural differences between a county and other surrounding areas, its cultural tourism resources may be more diversified due to the fact of cultural interaction, resulting in obvious differences in the types and quality of resources, while the high–high concentration distribution in geographical distribution is not significant. For example, the residents’ culture and customs in the Yazhou District of Sanya have formed unique cultural characteristics compared with other areas in Sanya. There are a large number of arcades distributed on Haikou, which form an arcade culture geographically, with a single cultural form and a high–high concentration of resources geographically.

#### 4.4.3. Regional policy

Regional policy is another important factor affecting the development of urban and rural tourism resources on islands. The mountainous area in the central part of Hainan Island is the main area in the urban–rural fringe, and it is also an underdeveloped area of regional tourism. The government chooses tourism resources in different regions as the first choice for development. Therefore, although the quantity and diversity of tourism resources in some urban–rural areas are similar, the spatial pattern of tourism resource quality is inconsistent. High–low and low–high concentrations of tourism resources were obvious in urban–rural areas, which means that the tourism development policies of neighboring administrative regions are different. For example, Diaoluo mountain spans Qiongzhong County and Lingshui County, and the Lingshui County government has focused its local tourism on mountain tourism resources. In contrast, the development of tourism at Diaoluo mountain in Qiongzhong County is insufficient, which leads to the unbalanced development of tourism resources of the same quality in different regions, and the resources in Qiongzhong County are now of low–low agglomeration.

#### 4.4.4. Level of public service facilities in rural

Last but not least, public facilities in rural areas have an important impact on the development of tourism. Transportation and accommodation facilities in mountainous areas are the foundation and supporting factors of tourism development. Improving the public infrastructure in rural areas, such as mountains and hills, can attract more tourists and earn more income. At the same time, data show that in 2018, Hainan Province received 67,4501 million tourists and realized a total tourism revenue of 81.199 billion yuan, while Hainan rural tourism realized a total of 10,2464 million tourists and a total rural tourism revenue of 3.216 billion yuan [43, 44]. It can be seen that tourism and rural tourism in Hainan Province are developing well, which also lays a good industrial foundation and development platform for tourism development in Hainan Province.

## 5. Analysis of the Hainan Island tourism development model

### 5.1. Graded development mode

There are differences in the quality of tourism resources in different regions, resulting in the spatial patterns of “high in the north, low in the south, high in the east and low in the west” tourism resource values on Hainan Island. High-level tourism resources are more likely to form tourism projects, while low-level tourism resources are more likely to form large-scale tourism projects. Low-level tourism resources can be combined with high-level resources to form a better tourism resources development zone [45, 46]. Therefore, according to the spatial distribution patterns of the tourism resources value grades on Hainan Island, different tourism development mode have been adopted.

1. The east coast of the northern plain area has become a priority development area, so it is necessary to intensify tourism development. The northern area should make full use of international cultural tourism resources, with Boao town as a representative, and accelerate integration with international tourism. The east coast has become tourism’s connecting line between Haikou in the north and Sanya in the south, while the eastern cities have become important tourism nodes and tourism industry clusters. The eastern coastline will continue to make full use of high-quality beach resources, giving full play to the advantages of coastal tropical holiday tourism and promoting the development of the tourism;
2. The west coast is a secondary tourism development zone. Compared with the west coast, the east coast has always been an important coastal tourism development area, relying on high-quality coastal resources and seawater quality. Tourism development on the west coast is underdeveloped, and tourism development basically follows the development of the east coast, developing beach tourism, but the quality of resources is far less than that of the east coast. Therefore, west coast tourism development should focus on its original characteristics. Compared with the eastern and central regions, the western resources are unique. Full use should be made of biological resources, tropical karst landforms, ports, and original ecological human resources, while paying attention to cultural excavation and protection and developing products of natural heritage, cultural heritage, and historical and cultural villages and towns;
3. The mountainous and hilly areas in the south-central are the last tourist areas to be developed. The Hainan government should work to protect resources, highlight the “tropical” characteristics, give full play to its resource advantages, and develop tropical forest tourism and holiday products such as exploration, rock climbing, and scientific research. It is also necessary to strengthen the construction of tourist traffic between mountain scenic spots and expanding viewing spaces. The southern mountainous areas are also a gathering place for ethnic minorities, so it is necessary to give full play to ethnic cultural characteristics and turn Tongshi town into a supporting city for tourism with ethnic characteristics.

### 5.2. Classified development mode

Based on the differences in the quantity, category, quality, and combination of tourism resources in the combined areas of Class I, Class II, Class III, and Class IV as well as the different economic development levels and tourism industry base of each township, Hainan Island was developed using a different combination of methods.

1. Class I combination areas should give full play to their advantages in the type diversity and resource combination, create product combination types from multiple dimensions, design and develop products that are combined with the tropical forest alone, national culture alone, or combined with nature and humanities;
2. Class II combination areas should give full play to their advantages in quantity and combination, develop horizontally in product combination, develop as many products of different combination types as possible to attract tourists, improve the length of time tourists stays, and transform tourism resources into tourism products to meet the diversified needs of tourists;
3. Class III combined zones have advantages in diversity and high-quality resources, and should focus on building high-quality resources, digging deep into the cultural connotations of the tourism resources, develop products vertically, build high-quality tourist attractions in depth, and become the key tourism areas in the region, thus promoting the high-quality development of the tourism industry;
4. Class IV combined areas should give full play to the advantages of abundant resources and carry out scale development and sustainable development of tourism resources in these areas.

### 5.3. Zoned development mode

Through the auto-correlation analysis of tourism resources value, it was concluded that the high–high tourism agglomeration areas and the low–low tourism agglomeration areas were in the north (Haikou area), and the low–low agglomeration areas included the south (Ledong–Baisha area), the west (Danzhou–Lingao area), and the middle (Qiongzhong area). Because of the differences in resource values, spatial relationships, and environmental characteristics, different types of tourism gathering areas adopt different development models.

1. The northern part (Haikou area) is rich in tourism resources, such as the coastline, craters, geology, Nanyang culture, historical sites, and villages, that produce the tourism value agglomeration effect in space. Therefore, we should keep the northern region fully open, dig deep into the cultural characteristics, and hold large-scale festivals, exhibitions, and sports events with wide influence in order to expand the influence of the northern region and enhance the overall international influence of Hainan Island;
2. Low–low agglomeration areas should rely on their own characteristics, fully tap the value of tourism resources, highlight characteristics, and pay attention to driving, regional linkage, and comprehensive development. Gathering areas in the south (Ledong–Baisha area) should make full use of mountain ecological resources, and develop tourism product clusters of “ecological leisure”, “ecological movement”, “ecological village”, “ecological sightseeing’, “ecological vacation”, and “ecological health preservation”. In the west (Danzhou–Lingao area), the treasure house of harbor and fishing village resources should be excavated, focusing on tropical rain forest sightseeing, exploration, rural vacations, and ethnic customs of harbor and fishing villages, with an emphasis on the dominant brand of “harbor and fishing village”, forming a series of products including landscape sightseeing, cultural experience, business meetings, and leisure vacation, and build a blue product combination brand. The central region (Qiongzhong region) should explore the cultural characteristics of the Li nationality, promote the innovation and upgrading of tourism products in the Qiongzhong region, build a tourism product system with its own characteristics, and build a characteristic product system of “theme scenic spots, characteristic towns, beautiful countryside, shared farms, characteristic homestays, ethnic trails, ethnic campsites, and the March 3 Limiao Cultural Festival”.

## 6. Conclusions and discussion

### 6.1. Conclusions

This study obtained a large sample of tourism resources data that covered natural and humanistic tourism resources from the perspective of tourism resource types. From a perspective of the state of development, it included not only the undeveloped but also the developed and developing states. On the basis of obtaining a large amount of individual spatial data and attribute information of tourism resources, this paper constructed an evaluation index system of regional tourism resources from four aspects: the quantity characteristics, quality characteristics, type characteristics, and spatial combination relationship, and then evaluated the tourism resources of each township, using a combination weighting method, and analyzed the spatial patterns of tourism resource values using the hierarchical clustering method and spatial autocorrelation method. On this basis, the spatial development mode of tourism resources were put forward. This research can determine the time sequence and mode of regional tourism resources development and provide spatial implications and suggestions for regional tourism planning and management. The main conclusions are as follows.

1. From the perspective of tourism resources combinations, this paper puts forward the ideas and methods of tourism resources evaluation. Taking the township scale as the spatial analysis unit, it constructed the regional tourism resources evaluation index system from four aspects: quantity, type, grade, and combination, and constructed the calculation method for the tourism resources value quality score using a combination weighting method;
2. According to the evaluation results, the overall value of Hainan Island’s tourism resources is low on the scale of villages and towns. The piedmont plain area in the north is higher than the hilly area in the south, and the eastern coastal area is higher than the western coastal area. From the four dimensions of quantity density, type diversity, grade superiority, and spatial combination, the distribution difference of tourism resources quality combination was obtained. The value of tourism resources on Hainan Island can be divided into four combined regions. The first type of combination area was diversified and well combined, which was characterized by small quantity but concentrated distribution. The second type of combination area was characterized by a large number and good combination, which was characterized by a small number and scattered distribution. The third type of combination area was diversity and high-quality resources, which was characterized by large number but scattered. The fourth type of combination area had a large number and concentration. From the analysis of spatial agglomeration effect, it was obvious that the value of tourism resources on Hainan Island is high and low. The results of high concentrations and low concentrations can be divided into four tourist resources gathering areas: north (Haikou area), south (Ledong–Baisha area), west (Danzhou–Lingao area), and the middle (Qiongzhong area);
3. On the basis of the evaluation’s results of the tourism resources quality and through the analysis of the spatial patterns of tourism resources quality, this paper puts forward three tourism development modes suitable for Hainan Island, including the grading, classification, and zoning development modes of tourism product design.

### 6.2. Discussion

#### 6.2.1. Innovation

In 2017, Haikou city in the north and Sanya city in the south received a total of 33,188 million tourists, accounting for 59.36% of the total annual tourism reception in Hainan Province. The total tourism income of the two cities was 67,216 billion yuan, accounting for 76.33% of the total tourism income for the province. Good locations, diversified tourism resources, and policy support were important factors for the strong momentum of tourism’s economic development in Sanya and Haikou. Wanning city, Qionghai city, and Lingshui County, as the eastern coastal areas, accounted for 12.14% of the province’s total tourism revenue in 2017, with obvious advantages in tourism economic development, and they gradually merged with Sanya and Haikou to form a marine tourism line along the eastern part of Hainan. In 2017, the total tourism economy of other regions was relatively small, and most of these counties and cities are concentrated in the central and western regions of Hainan Province. The gap in tourism development levels between the east and west coasts of Hainan Province is widening, which leads to the Matthew effect in regional tourism development. The Matthew effect leads to a development gap between active and lagging areas of tourism development in the region, which, in turn, means local governments are treated differently in terms of policy and resource enjoyment, and cross-regional enterprises cannot have an equal dialogue. In addition, community residents are dissatisfied that vested interests do not support tourism development. It can be seen that the influence of the Matthew effect on tourism development is extremely detrimental to overall regional tourism development [47]. It disturbs the normal tourism development order, destroys the economic harmony among different administrative regions, and leads to many regional public problems [48]. This study compared and analyzed the value of tourism resources on Hainan Island with the township administrative units as the research scale. The results were consistent with the differences in tourism development and distribution in Hainan Province, which indicates that the richness, hierarchical allocation, and regional combination of regional background resources are the basic factors restricting the development of regional tourism.

It has always been a challenge for decision makers when developing tourism policies to conduct scientific research at different levels and step by step in a region [49]. Scholars take a number of tourist areas which are then compared with each other as the evaluation object. First of all, they compare and evaluate the same type of tourism destinations in different regions and then evaluate the comprehensive potential of all different types of tourism destinations in each tourism region [50]. Stephen determined six time series of regional tourism development based on the model of county-level tourism resource [51]. Ge Jingfeng’s model for evaluating the quality potential of tourism land belongs to this type [52]. Wu established an evaluation model of tourism resources by using an index method [53]. Deng Yadong and others evaluated the opportunity and quality level of karst cave resources for tourism development in Yanjin Geopark [54]. In the above research, we mainly evaluated the development timing of tourist destinations including at the provincial scale and tourist areas and scenic spots. Our research takes villages and towns as the spatial unit of future tourism development, which is more microscopic than the county scale, which can not only reveal the spatial differences in tourism resource values within the region but also reflect the essence of regional tourism development at the rural–urban fringe. Therefore, the scale of villages and towns can better guide the region to follow a future tourism development strategy in an orderly way, realize the optimal layout of tourism resources in time and space, and improve land-use efficiency.

This study is different from traditional past studies, and the large sample size of tourism resource data obtained was one of the innovations of this paper, which is characterized by a large number of resources and full coverage of types. The second innovation was to quantitatively evaluate the quality of regional tourism resources from four aspects: quantity, quality, type, and combination, and to study the contribution mechanism of quantity, quality, type, spatial relationship, and distribution law of regional tourism resources to the quality of regional tourism resources. This study can not only scientifically and accurately determine the hot spots of resources but also dig deep into the spatial combination relationship among resources and determine the combination configuration and spatial patterns of tourism resources, which will play a guiding role in the key development and combination development of future tourism products.

#### 6.2.2. Implication

In this study, from the perspective of a “quality evaluation-spatial pattern-influence mechanism-development model”, the contribution mechanism of quantity, quality, type, spatial relationship, and distribution law of regional tourism resources to the value of regional tourism resources was analyzed using methods and technical means, such as model building and GIS spatial analysis, and the analysis framework and method system for evaluating the characteristics and relationship of regional tourism resources were put forward, enriching the research methods of tourism resources science and tourism planning. The results can determine the time sequence and layout of regional tourism resources development, and provide spatial decision support for the development and management of regional tourism resources.

#### 6.2.3. Limitations and future research

This study certainly has several limitations. Our research focused on the group value of several tourism resources in a region, that is, the value of regional tourism resources without considering the development conditions and ecological environment of the region such as attractiveness, accessibility, environmental quality, and tourism facilities. Saad Jubran H. Alkahtani conducted a systematic evaluation of the accessibility of the study area [55]. Qian Cheng evaluated the development potential of its low-carbon tourism resources by taking Xixi Wetland Park as an example [56]. In the future, in addition to the value of tourism resources, we can also consider a comprehensive evaluation of the development conditions and ecological environmental factors that affect the development of tourism.
